# Comparative Transcriptome Analysis of Defense Response of Potato to *Phthorimaea operculella* Infestation

**DOI:** 10.3390/plants12173092

**Published:** 2023-08-29

**Authors:** Chunyue Zhu, Xiaocui Yi, Miao Yang, Yiyi Liu, Yao Yao, Shengjiang Zi, Bin Chen, Guanli Xiao

**Affiliations:** 1 College of Agronomy and Biotechnology, Yunnan Agricultural University, Kunming 650201, China; zcynzb@163.com (C.Z.); 18287196028@163.com (X.Y.); m15350739150_2@163.com (M.Y.); 18313033732@163.com (S.Z.); booya611@163.com (Y.L.); yaoyao990926@163.com (Y.Y.); 2 College of Plant Protection, Yunnan Agricultural University, Kunming 650201, China

**Keywords:** plant–insect interactions, insect resistance-related pathways, mechanical damage, potato tuber moth herbivory stimulation

## Abstract

The potato tuber moth (PTM), *Phthorimaea operculella* Zeller (Lepidoptera: Gelechiidae), is one of the most destructive pests of potato crops worldwide. Although it has been reported how potatoes integrate the early responses to various PTM herbivory stimuli by accumulatively adding the components, the broad-scale defense signaling network of potato to single stimuli at multiple time points are unclear. Therefore, we compared three potato transcriptional profiles of undamaged plants, mechanically damaged plants and PTM-feeding plants at 3 h, 48 h, and 96 h, and further analyzed the gene expression patterns of a multitude of insect resistance-related signaling pathways, including phytohormones, reactive oxygen species, secondary metabolites, transcription factors, MAPK cascades, plant–pathogen interactions, protease inhibitors, chitinase, and lectins, etc. in the potato under mechanical damage and PTM infestation. Our results suggested that the potato transcriptome showed significant responses to mechanical damage and potato tuber moth infestation, respectively. The potato transcriptome responses modulated over time and were higher at 96 than at 48 h, so transcriptional changes in later stages of PTM infestation may underlie the potato recovery response. Although the transcriptional profiles of mechanically damaged and PTM-infested plants overlap extensively in multiple signaling pathways, some genes are uniquely induced or repressed. True herbivore feeding induced more and stronger gene expression compared to mechanical damage. In addition, we identified 2976, 1499, and 117 genes that only appeared in M-vs-P comparison groups by comparing the transcriptomes of PTM-damaged and mechanically damaged potatoes at 3 h, 48 h, and 96 h, respectively, and these genes deserve further study in the future. This transcriptomic dataset further enhances the understanding of the interactions between potato and potato tuber moth, enriches the molecular resources in this research area and paves the way for breeding insect-resistant potatoes.

## 1. Introduction

During long-term plant–insect interactions, plants have evolved complex defense strategies, including constitutive and inducible defense mechanisms to defend themselves against phytophagous insects [1,2]. Plants’ own inherent morphological structures or biochemical properties that resist insect feeding or pathogen invasion, such as waxy cuticles, glandular hairs, trichomes, spines, cell walls and lignin layers [3,4], as well as natural compounds in the body that sense and inhibit insects [5], all of which are categorized as constitutive defenses. Induced defenses are mostly activated only when the plant is exposed to external stimuli and play an important role in both direct and indirect ways for a short period of time [1,6]. In inducible defense, plants achieve direct defense against insects by producing toxic secondary metabolites (e.g., terpenoids, phenolic compounds, nitrogenous compounds such as alkaloids) and defense proteins (e.g., protease inhibitors PI, polyphenol oxidase, lectins, etc.), and or by reducing their own nutrient levels [1,7,8,9]. Indirect defense mainly refers to the release of pest-induced volatile chemicals (HIPVs) from plants that serve to attract predators of those herbivores to withstand insect infestation [10].

In general, after being subjected to insect feeding, the following steps occur in plants: (i) plant recognition of damage-associated and herbivore-derived molecular patterns (DAMPs and HAMPs) [11]; (ii) early signaling responses in plants; (iii) hormonal signaling responses; and (iv) reconfiguration of the transcriptome, metabolome and proteome [12]. Specifically, early signal events occurring before herbivore attack-related gene expression in plants mainly include ion imbalances, depolarization of membrane potential (Vm), variations of cytosolic Ca^2+^ concentrations, reactive oxygen species (ROS) burst and mitogen-activated protein kinases (MAPKs) signaling cascades [13]. Up to now, many HAMPs such as volicitin (termed 17-hydroxylinolenoyl-L-Gln, a fatty acid–amino acid conjugate (FAC) elicitor), califerins (the sulfooxy fatty acids), inceptin (a peptide fragment from chloroplast ATP synthase of cowpea plants digested by *Spodoptera frugiperda* larvae), a mucin-like protein (NlMLP) and glucose oxidase (GOX) have been isolated from insect oral secretions OS (e.g., regurgitant, saliva, insect-associated microorganisms including oral secretion bacteria) and interact with plant pattern recognition receptors PRRs (e.g., G-type lectin receptor kinases and leucine-rich repeat LRR-RK, etc.) [11,12]. The calcium ion is considered a second messenger in plant signaling pathways. A study has shown that rapid and highly-localized calcium elevations in Arabidopsis around the feeding sites of green peach aphid (*Myzus persicae*) [14]. Herbivorous insect Spodoptera littoralis feeding induced not only Ca^2+^ elevation in Arabidopsis and lima bean (*Phaseolus lunatus*) but also H_2_O_2_ production in lima bean leaves. Spodoptera littoralis infestation leaves have higher H_2_O_2_ concentrations when compared to mechanically damaged leaves [15,16]. Kandoth et al. demonstrated that the tomato MAPKs LeMPK1, LeMPK2, and LeMPK3 are important components of the systemin signaling pathway and are also required for successful defense against herbivorous insects in tomatoes [17]. There are close interactions between early defense signals related to plant–herbivore interactions [12,18]. Although some signal-transduction mechanisms are also evident in plant–pathogen interactions [13], further studies are required on the trade-offs between resistance to pathogens and herbivores in plants. Early signaling events are followed by changes in the signaling networks mediated by multiple hormones (such as JA, SA, ABA, and ETH), which in turn regulate downstream defense genes and the production of metabolites. Numerous studies have shown that JA plays a central role in regulating plant responses to phytophagous insects, while other hormones act in concert or antagonize the JA signaling pathway [1,12]. The selective activation of downstream-related pathways or signals by plants depends on the insect and plant species, and time differences in insect infestation can also lead to dramatic changes in plant defense responses [18,19,20]. Transcription factors play a key regulatory role in the global defense deployment of plants [12]. Rice transcription factor WRKY70 is required for protease inhibitor activation and resistance to herbivore *Chilo suppressalis* by positively regulating jasmonic acid (JA) biosynthesis, and WRKY70 enhances rice susceptibility to *Nilaparvata lugens* [21]. TFs regulate plant stress responses in an interactive manner by mediating secondary metabolism, such as the MYB-bHLH-WDR complex in Arabidopsis that regulates flavonoid biosynthesis [22].

As we mentioned earlier, early signaling events are rapidly activated thanks to the fact that plants can precisely perceive insect-derived elicitors (e.g., physical damage, insect oral secretions OS, ovipositional fluids, frass, etc.) [11,13]. Thus, at the plant–insect interface, mechanical damage and insect oral secretion stimuli OS are two main herbivory-associated stimuli [19,20]. Mechanical damage is often used as a control in experiments to investigate plant defense responses against herbivory. The results of microarray experiments in several plant species showed a huge overlap in gene expression patterns between mechanical damage and herbivore feeding, yet there are also transcriptional responses that are specific to both stimuli [1]. However, there are also examples that have demonstrated that gene expression and transcriptional profiles differ significantly between wounding alone and insect-induced activation, and saliva was identified as the agent responsible for the difference in the responses [23,24].

The potato, *Solanum tuberosum* L. (Solanales: Solanaceae), is one of the four major food crops, along with rice, wheat and maize. The potato is not only an essential vegetable crop and fodder crop but also an important raw material for the food industry, and its production is important for sustaining livelihoods and enhancing nutritional balance, especially in developing countries [25]. Potato tuber moth (PTM), *Phthorimaea operculella* (Zeller) (Lepidoptera: Gele-chiidae), which originated in Latin America, is an important pest of Solanaceae crops and is especially devastating to potatoes. Potato tuber moth larvae mine into the leaves and stems of potatoes or excavate tunnels into the tuber to feed, while PTM adults lay eggs in cracks on the surface of potato tubers, thus affecting potato growth and storage [26]. Due to the high reproductive potential of PTM, research on chemical and biological control has received much attention [27,28]. However, there is still a lack of green and effective control technology for this insect, such as improving potato resistance to this insect with the help of transgenic technology [29,30]. In this context, it is crucial to understand potato–moth interactions. Currently, there is a distinct lack of reported research on the molecular mechanisms of potato tuber moth feeding induced by potatoes at home and abroad. Therefore, in this study, we analyzed key signaling events occurring in potatoes in response to mechanical damage and PTM herbivory stress at multiple time points in the broad context of overall plant defense and compared the differences between the two stimuli, thus attempting to summarize the molecular mechanisms of potato under potato tuber moth feeding stress.

## 2. Results

### 2.1. Overview of Illumina Sequencing within Different Treatment Samples

To investigate the transcriptomic differences between mechanical damage and potato tuber moth (PTM) feeding at different time points, we performed RNA-Seq analyses on PTM-infested (P), mechanically damaged (M) and blank control (CK) potato plants.

The number of raw data obtained from 27 libraries (9 undamaged control for 3 h, 48 h and 96 h samples: CK3-1/CK3-2/CK3-3/CK48-1/CK48-2/CK48-3/CK96-1/CK96-2/CK96-3; 9 mechanical damage for 3 h, 48 h and 96 h samples: M3-1/M3-2/M3-3/M48-1/M48-2/M48-3/M96-1/M96-2/M96-3; 9 PTM infestation for 3 h, 48 h and 96 h samples: P3-1/P3-2/P3-3/P48-1/P48-2/P48-3/P48-1/P96-1/P96-2/P96-3) ranged from 37,271,202 to 51,880,920, and the number of clean data ranged from 37,224,532 (99.87%)~51,836,050 (99.91%). More than 81.9% of the clean data were successfully mapped to the reference genome of the potato, and the unique_mapped reads ranged from 14,048,153 (79.21%) to 42,974,467 (85.96%). The GC ratio ranged from 44.37% to 48.15%, and the Q30 values of these unique genes ranged from 91.08% to 94.09% (Table S1).

**Figure 1:**
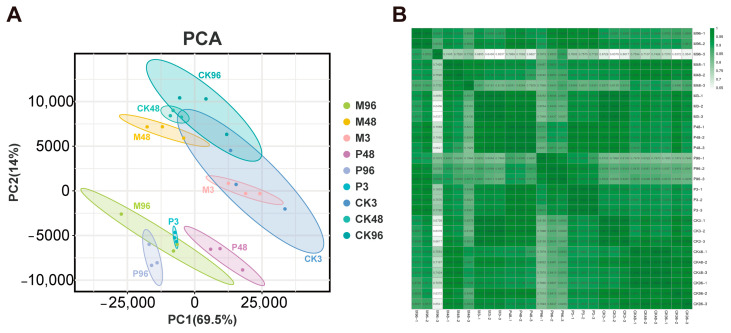
Global changes of transcriptome data for all treatment samples. (**A**) Sample principal component analysis diagram. The grouping patterns of samples were with respect to the first two principal components. A 69.5% variance is explained in PC1, and 14% variance is explained in PC2; the ellipse represents the 95% confidence interval of each treatment group. (**B**) Pearson correlation heatmap between different samples.

Principal component analysis (PCA) revealed that the gene expression profiles of PTM-infested plants were clearly separated from control plants, whereas the gene expression profiles of mechanically damaged plants partially overlapped with those of PTM-infested and control plants, which suggests principal component analysis (PCA) effectively separated the different transcriptome samples by treatments and PTM herbivory attack triggered stronger responses relative to mechanical damage (Figure 1A). PC1 accounting for 69.5% of the variation indicated a significant separation among PTM infestation for 3 h, 48 h and 96 h plants, whereas there was a weaker separation among mechanically damaged plants for 3 h, 48 h and 96 h along the PC2 axis (14% of the variance), and there was only a partially separation among control plants for 3 h, 48 h and 96 h, which suggests changes in transcriptomes could be linked to the developmental changes and stress response according to the time course within each treatment (Figure 1A). The correlation coefficients between different samples are shown in Figure 1B.

### 2.2. Identification and Functional Annotation of Differentially Expressed Genes (DEGs)

#### 2.2.1. Transcriptomic Changes in Response to Mechanical Damage (CK-vs-M)

**Figure 2:**
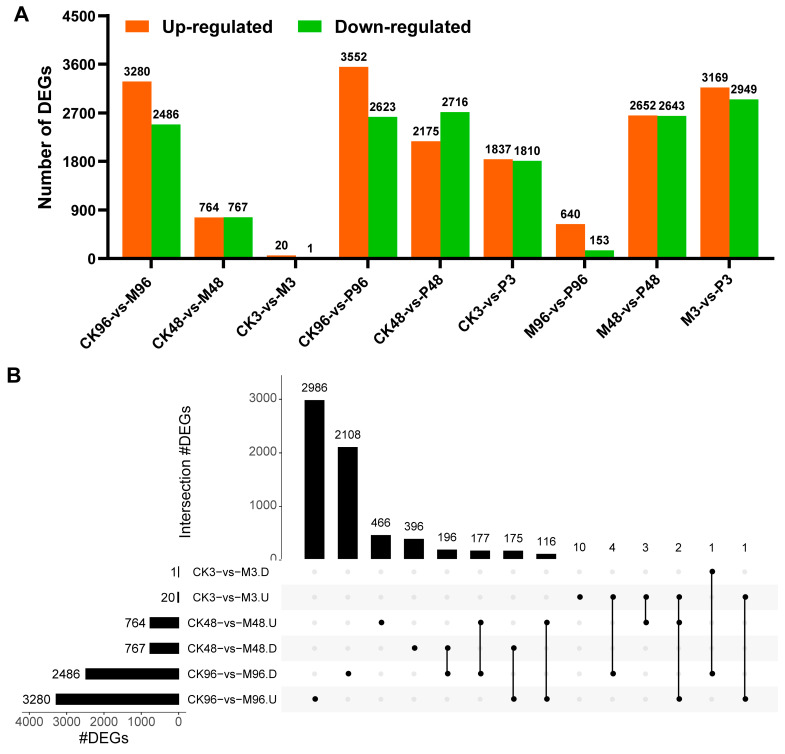
Overview of differentially expressed genes (DEGs) among different treatment comparisons for the same time points. (**A**) Number of up- and down-regulated differentially expressed genes (DEGs) among different comparisons at each time point. (**B**) UpSet intersection diagram illustrating the unique and shared DEGs between mechanical damage plants and control plants for 3 h, 48 h, 96 h. The intersection of shared (unique) DEGs are represented by a line connected to more plots (one plot), and the number of DEGs are shown on the top of the vertical bars. The black dots represent different comparison groups. U—up-regulated genes; D—down-regulated genes.

To understand the transcriptional changes in potato leaves after mechanical damage, transcription profiles of the mechanical wounding and control plants for 3 h, 48 h and 96 h were compared. Padj < 0.05 and an absolute value of Log_2_FoldChange (relative to the control) ≥1 was used as the standard for screening DEGs. A total of 1531 (764 up and 767 down) and 5766 (3280 up and 2486 down) DEGs were found for the comparisons: CK48-vs-M48 and CK96-vs-M96, respectively, whereas 21 (20 up and 1 down) DEGs induced only within CK3-vs-M3 comparison (Figure 2A). The total number of up-regulated genes (4064 DEGs) was more than that of down-regulated genes (3254 DEGs). The number of DEGs within intersections of CK3-vs-M3 (U/D) compared with other comparisons was less than 10 because of a small number (21 DEGs) of genes (Figure 2B). Within up-regulated genes, 466 and 2986 DEGs were uniquely expressed at 48 h and 96 h, respectively, and 116 DEGs were shared in common between two time points. A total of 396 and 2108 DEGs were uniquely expressed respectively within down-regulated genes after 48 h and 96 h, while 196 genes were common DEGs between the two time points (Figure 2B). The results indicated mechanical damage induced a significant transcriptome change in potato leaves through the time course.

GO term enrichment and KEGG pathway enrichment were performed on the total number of genes (21 DEGs for 3 h, 1531 DEGs for 48 h and 5766 DEGs for 96 h) after mechanical damage to predict their functions. The top 20 GO terms with the most significant enrichment including cellular components (CC), biological processes (BP) and molecular functions (MF), and pathways were presented in Supplementary Tables S2 and S3. Of the GO enrichment, the DEGs functions were related mainly to membrane (CC), negative regulation of biological process (BP), response to stimulus (BP), hydrolase activity (MF) and ion binding (MF) for 3 h, 48 h and 96 h. KEGG pathways were mainly associated with biosynthesis of secondary metabolites, protein processing in the endoplasmic reticulum and plant hormone signal transduction from 3 h to 96 h (Tables S2 and S3).

In addition, KEGG enrichment analysis was also performed for up- and down-regulated genes at 3 h, 48 h and 96 h after mechanical damage. Up-regulated genes were mainly concentrated in protein processing in the endoplasmic reticulum (48 h) and plant hormone signal transduction (3 h and 96 h) pathways, while down-regulated genes were mainly involved in DNA replication (48 h) and biosynthesis of secondary metabolites (96 h) Table S4).

#### 2.2.2. Transcriptomic Changes in Response to PTM Infestation (CK-vs-P)

**Figure 3:**
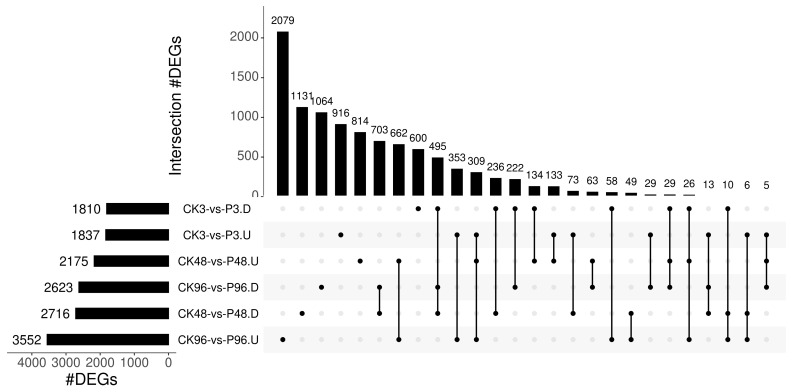
UpSet intersection plot of DEGs induced and suppressed by PTM infestation relative to the control of the same time. The intersections are shown with a line connected to one or more plots, and the number of common and unique DEGs is displayed above the vertical bars. The black dots represent different comparison groups. U—up-regulated genes; D—down-regulated genes.

The transcriptomes of PTM infestation and the undamaged control were compared to study the transcriptional changes induced by potato tuber moth feeding. The data exhibited 3647 (1837 up and 1810 down), 4891 (2175 up and 2716 down) and 6175 (3552 up and 2623 down) DEGs after 3 h, 48 h and 96 h, respectively. The number of DEGs induced by PTM infestation was more than that by mechanical damage at the same time, which suggested that PTM infestation triggered stronger responses than mechanical damage (Figure 2A). A total of 309 and 495 DEGs were continuously up-regulated and down-regulated for 3 h, 48 h and 96 h after PTM feeding. A total of 916, 814 and 2079 DEGs were uniquely up-regulated genes at 3 h, 48 h, and 96 h, which represent 49.9%, 37.4% and 58.5% of the total number of up-regulated DEGs at 3 h (*n* = 1837 DEGs), 48 h (*n* = 2175 DEGs) and 96 h (*n* = 3552 DEGs). 600, 1131 and 1064 genes were down-regulated DEGs uniquely for 3 h, 48 h and 96 h after insect herbivory (Figure 3).

The GO enrichment analysis of these DEGs characterized functions as: cell and cell part (CC), single-organism process (BP), transferase activity (MF). Ribosome, phenylpropanoid biosynthesis and carbon metabolism were the dominant KEGG enrichment pathways (metabolic pathways, biosynthesis of secondary metabolites and plant hormone signal transduction were excluded) (Tables S2 and S3).

After PTM infestation, the up-regulated DEGs functions were related mainly to ribosome (3 h), phagosome (48 h) and plant hormone signal transduction (96 h). Down-regulated DEGs were mainly associated with plant–pathogen interaction (3 h), ribosome (48 h), and protein processing in the endoplasmic reticulum (96 h) (Table S4).

#### 2.2.3. Transcriptomic Comparison Analysis between the Two Treatments (M-vs-P)

A total number of 12,206 DEGs were altered, of which 6118 (3169 up and 2949 down), 5295 (2652 up and 2643 down) and 793 (640 up and 153 down) DEGs were detected respectively for 3 h, 48 h and 96 h by comparing the transcription profiles of PTM-infested and mechanically damaged plants (Figure 2A).

**Figure 4:**
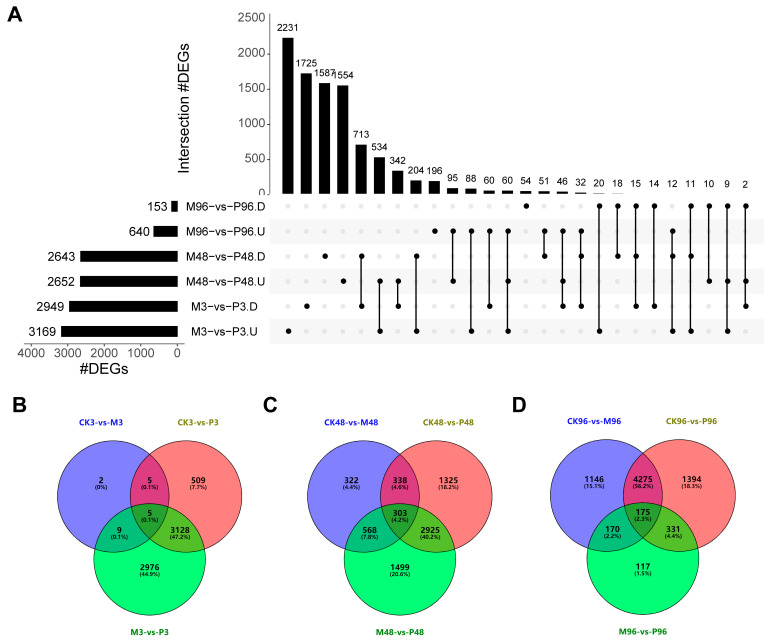
Comparative analysis of differentially expressed genes (DEGs) response to PTM infestation and mechanical wounding. (**A**) UpSet intersection diagram of up- and down-regulated DEGs from PTM-infested plants compared with mechanically-damaged plants at the same time. The black dots represent different comparison groups. U—up-regulated genes; D—down-regulated genes. (**B**–**D**) Venn diagram of differentially expressed genes (DEGs) that were common and unique among the CK, M, and P plant libraries after (**B**) 3 h, (**C**) 48 h, and (**D**) 96 h.

Interestingly, the least amount of DEGs was found at 96 h and the largest amount of DEGs was detected at 3 h within M-vs-P, which was the opposite of the case for CK-vs-M and CK-vs-P comparison groups, where the largest number of DEGs was found at 96 h, and the least number of DEGs was observed at three h. It is likely due to the potato leaves the internal state of insect feeding for 96 h is similar to that of mechanical damage for 96 h, which is consistent with the results of PCA data. 2231 DEGs, 1554 DEGs and 196 DEGs occurred uniquely, respectively, for the comparisons: M3-vs-P3. U, M48-vs-P48. U and M96-vs-P96. U, while 60 DEGs were common genes among three comparisons (Figure 4A). 1725, 1587 and 54 DEGs were uniquely expressed respectively within three comparison groups: M3-vs-P3. D, M48-vs-P48. D and M96-vs-P96. D, whereas only 15 DEGs were shared in common among three comparisons (Figure 4A).

GO enrichment analysis showed genes functions related mainly to plastid (CC), single-organism metabolic process (BP), oxidoreductase activity (MF) and catalytic activity (MF). KEGG analysis of genes comprising M-vs-P groups were enriched in pathways related to MAPK signaling pathway-plant (3 h), biosynthesis of amino acids (48 h), and biosynthesis of secondary metabolites (96 h). The detailed information of KEGG enrichment terms for up- and down-regulated genes in the M-vs-P comparison groups were presented in Supplemental Table S10. Functional analysis results of the M-vs-P were similar to the CK-vs-P comparisons groups (Tables S2 and S3).

In addition, the distribution of the abundances of transcripts among CK, M and P plant libraries were also compared strategically according to the method from Wang Dan et al. [19]. Venn diagrams revealed that 2976, 1499 and 117 DEGs were expressed only in M-vs-P comparisons for 3 h, 48 h and 96 h, respectively (Figure 4B–D). These DEGs may be related to other potato tuber moth herbivorous stimuli (e.g., insect oral secretions OS, walking, frass depositions, ovipositional fluids, etc.), which requires further validation through experiments.

### 2.3. Expression Analysis of DEGs Involved in Plant Hormone Biosynthesis

When plants are subjected to external stimuli (mechanical damage, herbivores attack, pathogen infection and abiotic stress), phytohormones can accumulate as signaling molecules, and induce the expression of downstream defense genes to activate the immune system against environmental stresses. In terms of JA, SA, ET and ABA biosynthesis pathways related to plant resistance, potato plants had different phytohormonal responses to external stress across the time course [18]. The expression level and functional annotations of related genes were presented in Supplementary Table S5.

**Figure 5:**
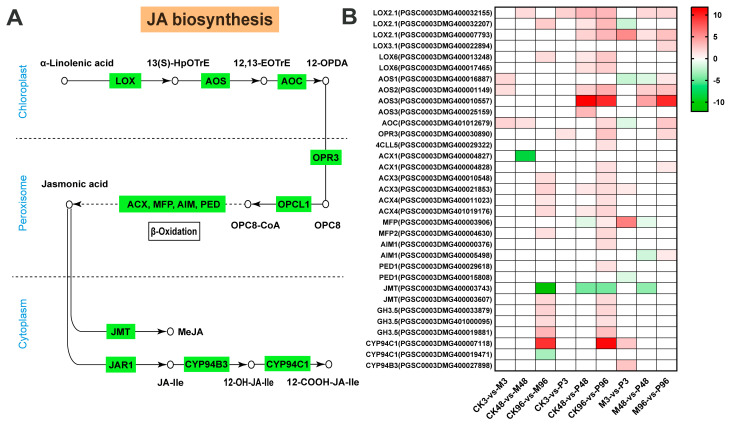
Analysis of jasmonic acid (JA) pathway gene expression. (**A**) Schematic diagram of the JA biosynthesis pathway. The circles represent the production, the green rectangles represent the genes that encoded for biosynthetic enzymes. LOX—lipoxygenase; AOS—allene oxide synthase; AOC—allene oxide cyclase; OPR3—12-oxophytodienoate reductase; OPCL—4-coumarate-CoA ligase; ACX—acyl-coenzyme A oxidase; MFP—enoyl-CoA hydratase/3-hydroxyacyl-CoA dehydrogenase; AIM—Fatty acid beta-oxidation multifunctional protein; PED—3-ketoacyl-CoA thiolase; JMT—JA carboxyl methyltransferase; JAR1—jasmonate resistant 1; CYP—cytochrome P450; 13(S)-HpOTrE— (9Z,11E,15Z)-(13S)-13-Hydroperoxyoctadeca-9,11,15-trienoic acid; 12,13-EOTrE—12,13-Epoxyoctadecatrienoic acid; 12-OPDA—12-oxo-phytodienoic acid; OPC8—3-oxo-2-(2′(Z)-pentenyl)-cyclopentane-1 octanoic acid; OPC8-CoA—3-oxo-2-(2′(Z)-pentenyl)-cyclopentane-1 octanoic acid coenzyme A; MeJA—methyl jasmonate; JA–Ile—jasmonoyl-isoleucine. (**B**) Heatmap of JA biosynthesis-related gene expression. Red represents up-regulated genes and green represents down-regulated genes. Color coding represents the range of log2 (foldchange relative to control). GH3.5—indole-3-acetic acid-amido synthetase GH3.5.

α-linolenic acid (18:3) is a precursor of jasmonic acid (JA) synthesized from the octadecanoid pathway occurring in the chloroplast, peroxisome and cytoplasm sequentially in plants [31]. In the potato, LOX enzymes are divided into 9-LOX in cytosol and 13-LOX in chloroplast depending on the oxidation position of α-linolenic acid [32]. In terms of α-linolenic acid metabolism pathway, 9-LOX genes leading to the production of 10-oxo-11,15-phytodienoic acid (10-OPDA) were not found, whereas six 13-LOX genes related to the formation of 12-oxo-phytodienoic acid (12-OPDA) were up-regulated within all comparisons, except for the down-regulated gene encoding LOX2.1 (PGSC0003DMG400032207) in M3-vs-P3 (Figure 5, Table S4). The LOX product (9Z,11E,15Z)-(13S)-13-Hydroperoxyoctadeca-9,11,15-trienoic acid (13(S)-HpOTrE) was converted progressively by AOS and AOC to the final product 12-OPDA of the plastid-located part of JA biosynthesis. Subsequently, 12-OPDA is catalyzed gradually by OPDA reductase (OPR3) and OPC-8:0 CoA ligase 1 (OPCL1), resulting in the JA production ultimately according to β-oxidative steps in peroxisome [33]. Most of the genes encoding AOS, AOC, OPR3 and OPCL1 were up-regulated between all comparisons, whereas one AOS gene was down-regulated in 3 h or 48 h P/M comparisons, and one AOC gene was also down-regulated at 3 h P/M comparison. Among genes related to the enzymes of fatty acid β-oxidation, ACX1(PGSC0003DMG400004827) in CK48-vs-M48 and AIM1 (PGSC0003DMG400005498) in M48-vs-P48 had a low expression, respectively. MFP (PGSC0003DMG400003906) was down-regulated at 48 h both in P/CK and P/M comparisons, and PED1(PGSC0003DMG400015808) was also down-regulated in M3-vs-P3 comparisons (Figure 5, Table S5).

In the cytoplasm, JA is metabolized into different structures by various chemical reactions, such as methyl jasmonate (MeJA), jasmonoyl-isoleucine (JA–Ile), cis-jasmone (CJ) and 12-hydroxyjasmonic acid (12-OH-JA). Among all genes, JMT (PGSC0003DMG400003743) had the lowest FC value in CK96-vs-M96, and remained down-regulated in CK48-vs-P48, CK96-vs-P96 and M48-vs-P48 comparisons, while the other JMT gene had a weak induction at 96 h M/CK and P/CK. Except for the CYP94C1(PGSC0003DMG400019471), all genes encoding JAR, CYP94B3 and CYP94C1 were up-regulated. The above results indicated that the DEGs related to MeJA production were inhibited, whereas JA–Ile synthesis was strongly induced (Figure 5, Table S5).

Overall, among the 33 JA synthetic pathway genes, there were 3 (3 up and 0 down), 3 (2 up and 1 down) and 14 (12 up and 2 down) DEGs detected in the potato at 3 h, 48 h and 96 h after mechanical damage, respectively. A total of 2 (2 up and 0 down), 12 (10 up and 2 down) and 25 (24 up and 1 down) DEGs after PTM infestation were observed for three time points, respectively. In P/M comparisons, there were 9 (5 up and 4 down) DEGs for 3 h, 8 (4 up and 4 down) DEGs for 48 h and 10 up-regulated genes for 96 h, respectively (Figure 5, Table S5). Results suggest that mechanical injury and PTM feeding lead to the up-regulation of most JA biosynthesis-related genes, and more DEGs are detected according to the time course.

Plants synthesize salicylic acid through two pathways: the isochorismate (ICS) pathway and the phenylalanine ammonia-lyase (PAL) pathway [34]. Chorismate is required as the common precursor in both pathways. In the ICS pathway, chorismate is catalyzed by ICS enzymes to produce isochorismate, which are transported from plastids to cytosol through EDS localized on chloroplast envelope and subsequently was conjugated with glutamate via the PBS3 to produce isochorismate-9-glutamate (IC-9-Glu). Ultimately salicylic acid was produced via spontaneous decay of IC-9-Glu or catalytic reaction of IC-9-Glu by EPS1. In the other pathway, chorismate was catalyzed to produce phenylalanine in chloroplasts, which entered the cytoplasm and was converted by PALs to produce trans-cinnamic acid. After entering the peroxisome, trans-cinnamic acid is catalyzed to be benzoic acid, resulting in the production of salicylic acid finally in cytosol [34,35].

**Figure 6:**
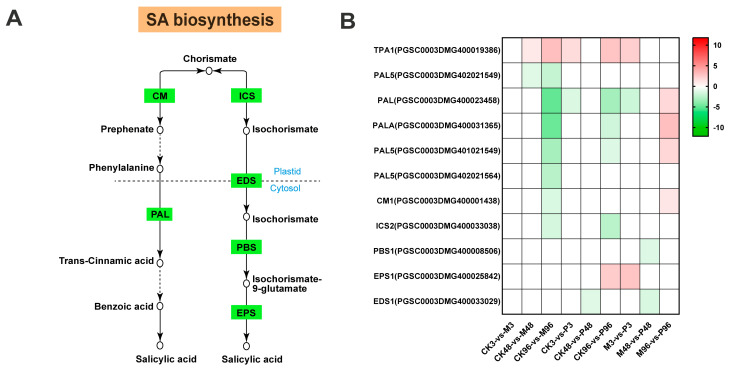
Analysis of salicylic acid (SA) pathway gene expression. (**A**) Schematic diagram of the SA biosynthesis pathway. The circles represent the production, the green rectangles represent the genes that encoded for biosynthetic enzymes. CM—chorismate mutase; PAL— phenylalanine ammonia lyase; ICS—isochorismate synthase; EDS—enhanced disease susceptibility; isochorismate is transported by the multidrug and toxin extrusion (MATE) transporter EDS from plastid to the cytosol. PBS—avr PphB susceptible; EPS—enhanced pseudomonas susceptibility. (**B**) Heatmap of JA biosynthesis-related gene expression. Red represents up-regulated genes and green represents down-regulated genes. Color coding represents the range of log2 (foldchange relative to control).

In terms of the DEGs encoding phenylalanine ammonia lyase (PAL), five DEGs were down-regulated among the first seven comparisons, whereas the opposite was the case for the TPA1 gene where the gene was up-regulated among these comparisons. Notably, all the three PAL genes detected in M96-vs-P96 comparison were up-regulated. The expression of the gene encoding chorismate mutase (CM) was similar to the gene encoding PAL (Figure 6, Supplemental Table S5). Among the genes encoding ICS, EDS, PBS and EPS, only EPS1 (PGSC0003DMG4000025842) was up-regulated in CK96-vs-P96 and M3-vs-P3 comparisons; other genes were down-regulated. Among the total of 11 up-regulated genes in 9 comparisons groups, there were 5 genes with high fold change (FC > 5), 1 gene with FC > 4, 3 genes with FC > 3, 2 genes with FC > 2, which suggested only several genes involved in SA biosynthesis pathway were highly induced in response to two treatments (Supplemental Table S5).

**Figure 7:**
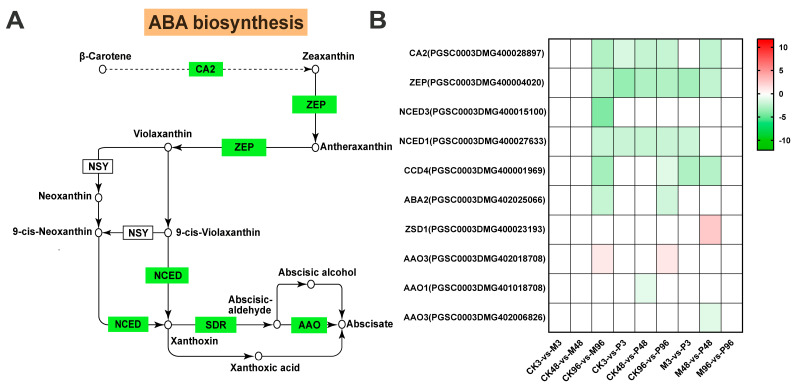
Analysis of abscisic acid (ABA) pathway gene expression. (**A**) Schematic diagram of the ABA biosynthesis pathway. The circles represent the production, the green rectangles represent the genes that encoded for biosynthetic enzymes. CA2—beta-carotene 2-hydroxylase; ZEP—zeaxanthin epoxidase; NSY—neoxanthin synthase; NCED—9-cis-epoxycarotenoid dioxygenase; SDR—short-chain dehydrogenase/reductase; AAO—abscisic-aldehyde oxidase. (**B**) Heatmap of ABA biosynthesis-related gene expression. Red represents up-regulated genes, and green represents down-regulated genes. Color coding represents the range of log2(foldchange relative to control). CCD4—carotenoid cleavage dioxygenase; ABA2—xanthoxin dehydrogenase; ZSD1—secoisolariciresinol dehydrogenase.

The currently accepted ABA biosynthesis pathway in plants is from the oxidative cleavage of carotenoids. β-carotene is utilized via various enzymes to produce trans-isomer zeaxanthin [36]. The conversion of zeaxanthin to violaxanthin catalyzed by zeaxanthin epoxidase (ZEP) within a two-step epoxidation via antheraxanthin is the formal first step of ABA biosynthesis [37]. Subsequently, violaxanthin was converted by neoxanthin synthase (NSY) and an unknown isomerase according to the two pathways to generate 9′-cis-neoxanthin and 9′-cis-violaxanthin [38]. The committed step of ABA biosynthesis is that 9′-cis-epoxycarotenoid dioxygenase (NCED) enzyme family split the cis-xanthophylls (9′-cis-violaxanthin and 9′-cis-neoxanthin) to synthetize C15 xanthoxin [39]. The above steps are carried out in plastids, and the next steps of the conversion from xanthoxin to ABA take place in the cytosol. The xanthoxin is converted into abscisic aldehyde (ABAld) utilizing a short-chain alcohol dehydrogenase (SDR), which is eventually oxidized to be abscisic acid (ABA) by abscisic-aldehyde oxidase (AAO) [40,41]. In addition, xanthoxin can also first be catalyzed to xanthoxic acid by AO isoform(s) and then xanthoxic acid is converted to ABA. The minor shunt pathway involving abscisic aldehyde as an intermediate is also an important source of ABA [42]. Overall, most of the genes involved in the ABA biosynthesis pathway in all comparisons were clearly down-regulated. Only one gene encoding ZSD1(PGSC0003DMG400023193) with FC > 5 was highly expressed, while two gene encoding AAO3 (PGSC0003DMG402018708) with FC > 2 were low expressed both in CK96-vs-M96 and CK96-vs-P96 comparisons (Figure 7 and Table S5).

**Figure 8:**
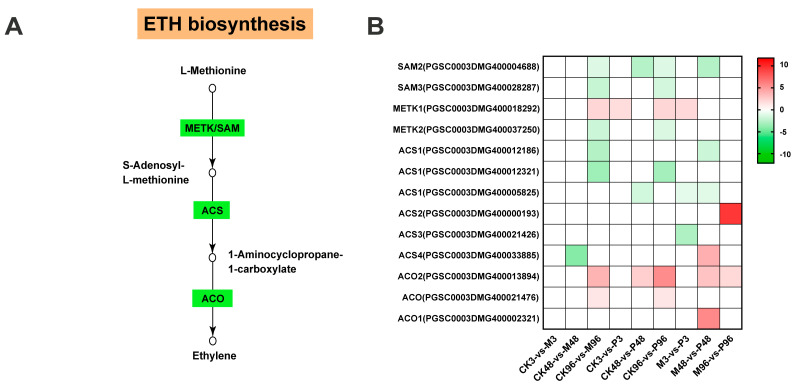
Analysis of ethylene (ET) pathway gene expression. (**A**) Schematic diagram of the ET biosynthesis pathway. The circles represent the production, the green rectangles represent the genes that encoded for biosynthetic enzymes. METK/SAM—s-adenosylmethionine synthase; ACS—1-aminocyclopropane-1-carboxylate synthase; ACO—1-aminocyclopropane-1-carboxylate oxidase. (**B**) Heatmap of ET biosynthesis-related gene expression. Red represents up-regulated genes and green represents down-regulated genes. Color coding represents the range of log2 (foldchange relative to control).

Compared with other plant hormones, the biosynthesis of ethylene is relatively simple. The first step is that L-methionine as the substrate was converted by S-adenosylmethionine synthase (S-AdoMet or SAM synthase) to generate S-adenosyl methionine (S-AdoMet or SAM) [43]. The subsequent step of S-AdoMet converted to 1-aminocyclopropane-1-carboxylate (ACC) utilizing ACC synthase (ACS) is the rate-limiting step [44]. Eventually, ACC was catalyzed by 1-aminocyclopropane-1-carboxylate oxidase (ACO) to produce ethylene [45]. Among the 13 genes of ethylene biosynthesis pathway, mechanical damage induced a total of 3 up-regulated gene expressions and PTM infestation induced a total of 5 up-regulated gene expressions in the corresponding treatment groups. A total of six up-regulated genes appeared in M-vs-P comparisons. Among the 11 up-regulated genes, there were a total of 2 genes with FC > 2, 5 genes with FC > 3, 1 gene with FC > 4, 1 gene with FC > 5, 2 genes with FC > 10, 2 genes with FC > 40 and 1 gene with FC > 500, which suggested ethylene pathway were highly induced locally (Figure 8 and Table S5).

### 2.4. Expression Analysis of DEGs Involved in Reactive Oxygen Species (ROS) Signaling

Reactive oxygen species (ROS) mediate the normal metabolic and developmental processes in plants as key signaling molecules and enable plants to respond quickly to exogenous abiotic and biotic (including different pathogens and pests) stress [46]. We investigated genes encoding the key enzymes involved in metabolism reactions related to ROS production and scavenging in different comparisons. The gene expression of each comparison is shown in Supplemental Table S6.

A total of 13 DEGs (9 up and 4 down) and 20 DEGs (8 up and 20 down) related to ROS generation were observed at 48 h and 96 h post mechanical damage, respectively, whereas there was no DEGs occurring at 3 h in potato leaves. PTM infestation induced 21 DEGs (11 up and 10 down) for 3 h, 25 DEGs (12 up and 13 down) for 48 h and 27 DEGs (13 up and 14 down) for 96 h associated with ROS production, respectively. Compared with mechanical damage, PTM infestation triggered more up- and down-regulated DEGs at the same time point. Within comparisons of PTM infestation relative to mechanical damage, 30 DEGs (16 up and 14 down), 38 DEGs (18 up and 20 down) and 10 DEGs (8 up and 2 down) were found at 3 h, 48 h and 96 h, respectively (Table 1, Supplemental Table S6). The genes encoding acyl-CoA oxidase (ACX) had the largest group of the up-regulated genes within M/CK and P/CK comparisons, respectively, while the number of amine oxidase (AO) genes and gibberellin dioxygenase (GAOX) genes within down-regulated genes was the largest group in two comparisons.

In terms of the genes encoding ROS scavenging, CK-vs-M groups comprised 24 DEGs (14 up and 10 down) and 59 DEGs (20 up and 39 down) expressed at 48 h and 96 h, respectively. A total of 35 DEGs (13 up and 22 down), 78 DEGs (51 up and 27 down) and 73 DEGs (46 up and 27 down) were identified at three time points after PTM infestation, respectively. A total of 15, 54, 32 up-regulated genes and 64, 38, 3 down-regulated genes were found respectively at 3 h, 48 h and 96 h within M-vs-P comparisons (Table 2, Supplemental Table S6). The total number of up-regulated genes encoding peroxidase (POD) was the largest group, whereas glutathione s-transferase (GST) had the opposite gene expression with the largest down-regulated group among all comparisons (Table 2).

### 2.5. Expression Analysis of DEGs Involved in Transcription Factors (TFs)

**Figure 9:**
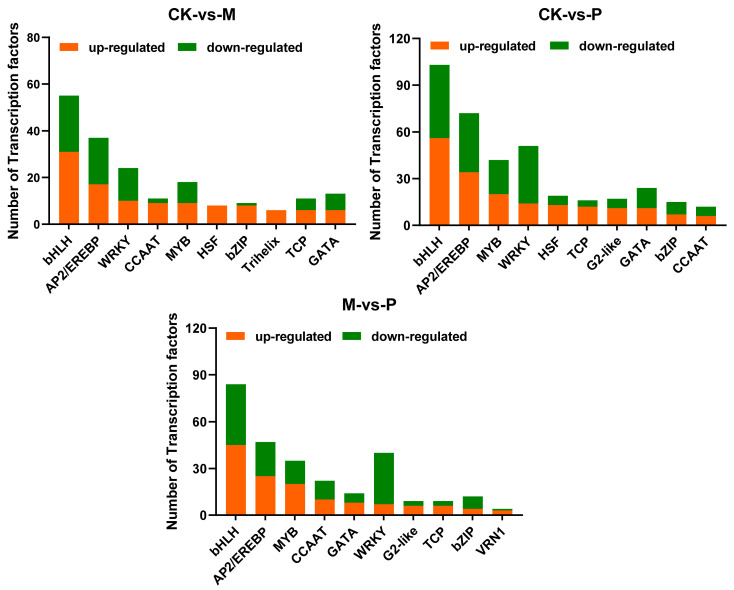
The total number of the top 10 transcription factors (TF) families with the largest number of up-regulated genes among the CK-vs-M, CK-vs-P and M-vs-P comparisons.

Currently, there are about 58 transcription factors (TFs) families existing in higher plants, which are important components in regulating the perception of external stimuli signals and the expression of corresponding stress-responsive genes [47]. The detailed information on TFs genes in each comparison was presented in Supplemental Table S7. The numbers of up- and down-regulated TFs genes from 3 h to 96 h in CK-vs-M, CK-vs-P and M-vs-P comparisons were added up, respectively, and the top 10 TFs families with the largest number of up-regulated genes are shown in Figure 9. There were 1 (1 up and 0 down), 55 (21 up and 34 down) and 163 (104 up and 59 down) DEGs that appeared at three time points, respectively, in CK-vs-M comparison and the top 10 TFs families (192 genes) accounted for more than 87% of these genes (219 genes) (Table S7). In CK-vs-P comparisons, we identified 38, 52 and 112 up-regulated genes as well as 36, 92 and 78 down-regulated genes at 3 h, 48 h and 96 h, respectively. In P/M comparison, 135 DEGs (69 up and 66 down), 144 DEGs (58 up and 86 down) and 33 DEGs (21 up and 12 down) were detected at three time points, respectively. The top 10 TFs families, respectively, accounted for more than 90% of CK-vs-P comparison (408 genes) and 88% of M-vs-P comparison (312 genes) (Table S7).

Among the top 10 TFs families, 9 TFs families, including bHLH, AP2/EREBP, WRKY, CCAAT, MYB, HSF, bZIP, TCP, and GATA, were commonly identified in three comparison groups and may have overlapping effects in mediating the defense responses to PTM infestation and mechanical damage (Figure 9). However, the difference is that the Trihelix family and G2-like family only appeared, respectively, in the CK-vs-M and CK-vs-P comparisons, which suggested the Trihelix family was mainly involved in defense responses to mechanical damage, while the G2-like family mainly mediated defense responses against PTM infestation (Figure 9). The dominant defensive role of the G2-like family against P treatment has also been demonstrated in the P/M comparison (Figure 9).

The up- and down-regulated transcription factor-related genes in CK-vs-M, CK-vs-P and M-vs-P comparisons were analyzed by KEGG. The results showed that up-regulated genes in CK-vs-M and CK-vs-P comparisons were enriched in circadian rhythm, MAPK signaling pathway and plant–pathogen interaction. The down-regulated genes of CK-vs-M are mainly related to protein processing in the endoplasmic reticulum, while the down-regulated genes of CK-vs-P are mainly associated with plant hormone signal transduction (Supplemental Table S8).

### 2.6. Expression Analysis of DEGs Involved in Plant Secondary Metabolites (PSMs)

Plant secondary metabolites have essential roles in the regulation defense signalings and function in the plant defense system against herbivore attacks and harsh environments [48]. The detailed information on PSMs-related DEGs are shown in Supplemental Table S9. The numbers of up-regulated genes related flavonoid biosynthesis were much smaller than that of down-regulated genes in a majority of comparisons, which indicated that the biosynthesis of flavonoids may have been inhibited in the process of plant resistance to two treatments. Compared to flavonoids, the biosynthesis of terpenoids in potatoes was gradually induced by PTM feeding from 3 h to 96 h (Table 3 and Table S9). In CK-vs-M comparisons, there was no one up-regulated gene related alkaloids and steroids biosynthesis appeared for first two time points, which showed alkaloids and steroids were not involved in response against mechanical damage at 48 h post mechanical damage. Interestingly, 17 (6 up and 11 down), 24 (15 up and 9 down) and 25 (12 up and 13 down) DEGs related to alkaloids as well as 8 (5 up and3 down), 8 (4 up and 4 down) and 14 (9 up and 5 down) DEGs associated with steroids biosynthesis were detected at three time points respectively in CK-vs-P comparisons, which suggested that alkaloids and steroids have been involved in defense reactions of potato to PTM at 3 h and both remained until 96 h post-infestation. Compared with other PSMs, only several up-regulated genes were identified in each comparison, which showed that the potato may induce low concentrations of quinones in response to PTM feeding and mechanical damage (Table S9).

### 2.7. Expression Analysis of DEGs Involved in Plant–Pathogen Interactions and Defense Response

The incursion of pests and pathogens can elicit the up-regulation of related plant defensive gene families, including plant–pathogen interaction genes, lectin, proteinase inhibitors, chitinases and MAPK cascades signaling genes [49]. Detailed information about the various defense genes are presented in Tables S10 and S11.

PIs and CHIs both belong to pathogenesis-related (PR) proteins [50]. Proteinase inhibitors (PIs) families are a large and complex group of plant proteins and have the ability to form complexes with a widely accepted five classes of proteolytic enzymes, including serine, cysteine, threonine, aspartic and metallo-proteases, which suppress the normal assimilation of food proteins and bring the adverse effects for insects [51]. In our study, a total of five serine-, four cysteine-, three aspartic-type protease inhibitor-related genes and two protease inhibitor-related proteins were identified (Table S11). A majority of genes (50 genes) encoding PIs were up-regulated. The largest number of up-regulated genes both appeared at 48 h post two treatments relative to control treatments (Table 4).

Plant chitinase (CHIs) can attack chitin as the structural molecule in skeletons of insects as well as cell walls of fungi and hence chitinases play a potential role against pathogens and insects [52]. In CK-vs-M comparisons, up/down-regulated genes related to CHIs both appeared at 48 h and the number both peaked at 96 h. The number of up-regulated genes (one gene) were induced at 3 h by PTM infestation and the number (six genes) peaked at 96 h, while the down-regulated genes were the opposite case, maximum number of genes (seven genes) detected at 3 h and minimum number of genes (one gene) detected at 96 h (Table 4).

MAPK cascades serve as an important element for transcriptional activation of herbivore defense-related genes and the accumulation of defensive metabolites [4,53]. The MAPK-related up/down-regulated genes post two treatments showed similar expression patterns, with the largest number of genes appearing at 96 h (Table 4).

**Figure 10:**
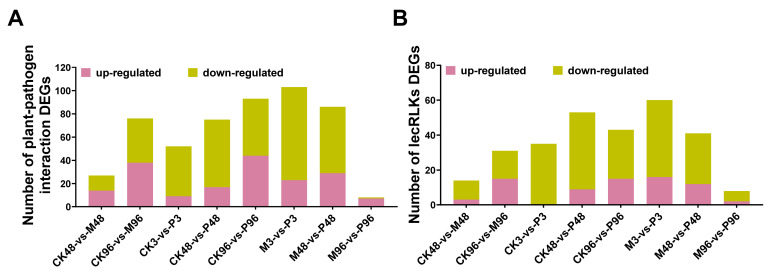
Genes differentially expressed in all comparisons. (**A**) The number of up- and down-regulated genes associated with plant–pathogen interactions. (**B**) The number of up- and down-regulated genes related to lectin receptor-like protein kinases (LecRLKs).

Previous studies showed that the gene expression level of attack from insect herbivory and infection by pathogens can have a partial or considerable overlap [54,55]. LecRLKs (possessing three subclasses: L-, G-, and C-type LecRLKs), as a subfamily of receptor-like protein kinases, consist of three domains: extracellular lectin domain, intermediate transmembrane domain and intracellular kinase domain, which play crucial roles in development, stress perception and pathogen detection [56,57]. The up-regulated DEGs involved in plant–pathogen interaction DEGs sequentially accounted for 51.8%, 50%, 17.3%, 22.7%, 47.3%, 22.3%, 33.7% and 87.5% of the total number of DEGs of each comparison (CK3-vs-M3 comparison was excluded). GO analysis of each comparison group showed that the up-regulated gene function at 48 h post-mechanical damage were mainly associated with response to stimulus (BP), cellular protein metabolic process (BP) and protein binding (MF). The up-regulated genes of PTM infestation for 48 h were mainly enriched in single-organism process (BP) and catalytic activity (MF). However, compared with the control, the enriched GO terms of up-regulated genes related to plant–pathogen interaction were similar at 96 h post two treatments (Supplemental Table S12). The up-regulated DEGs screened from lecRLKs-related genes, respectively, accounted for 21.4%, 48.4%, 0%, 17.0%, 34.9%, 26.7%, 29.3% and 25.0% of total number of DEGs of each comparison (except for CK3-vs-M3 comparison) (Figure 10). This indicates that the number of up-regulated DEGs increased over time after two treatments. The GO analysis of up-regulated lecRLKs genes comprising each comparison group was mainly enriched in transferase activity (MF), kinase activity (MF), catalytic activity (MF) and biological processes (BP) (Supplemental Table S13).

## 3. Discussion

Currently, the research about the defense mechanisms of the potato in response to *Phthorimaea operculella* infestation has remained limited. A previous study has revealed how potato plants integrate accumulatively a multitude of herbivory components from potato tuber moth and show the early defense responses of potato leaf to these reorganized stimuli [56,57]. In our study, the global transcriptomic response of potato plants to PTM infestation and mechanical damage was monitored by RNA-Seq in CK, M, P plants harvested at 3 h, 48 h and 96 h. Overall, the transcriptomes were significantly remodeled under the PTM attack and mechanical damage during the time course.

Except for the GO terms in common between mechanical damage and PTM infestation across three time points, two treatments at 3 h mainly activated the secondary metabolism of the potato, such as the S-glycoside and indoleacetic acid metabolic process (CK3-vs-M3) as well as the flavonoid metabolic process (CK3-vs-P3) (Table S2). At 48 h, GO terms included negative regulation of cellular metabolic process (CK48-vs-M48) and regulation of multicellular organismal development (CK48-vs-P48), which indicated two treatments mainly suppressed the growth and development of potatoes. At 96 h, there were significant enrichment in carbohydrate and polysaccharide metabolic process in M/CK comparison as well as organic acid transmembrane transport in P/CK comparison (Table S2), indicating the two treatments resulted in the redistribution of energy in potato plants. Physical damage can lead to the decline of the photosynthesis ability of plants and a decrease in ATP production by photosynthesis phosphorylation, resulting in energy deficiency [18]. Compared with mechanically wounding, insect feeding caused more severe damage to potato plants, which prompted the potato to obtain energy for growth and defense through other ways, such as organic acid metabolism.

The stimuli from insect feeding can be roughly divided into several types: mechanical damage, oral secretion stimuli OS, walking, frass depositions, and ovipositional fluids, etc. [19]. In M-vs-P, we identified different numbers of DEGs induced only by mechanical damage and PTM infestation, as well as some DEGs that may come from other potato tuber moth herbivorous stimuli by strategic comparative transcriptome analysis (Figure 4B–D). These genes may be key components and factors that determine the different gene expression patterns of downstream insect defense-related signaling pathways.

There were 11 up-regulated DEGs associated with ROS generation found at 3 h after PTM infestation and the number of genes was continuously increasing until 96 h (Table 1).

In the study of Mao et al. [20], ROS signaling was activated by PTM-derived herbivory-related cues as early as 1 h after treatment. In general, the process of ROS generation occurs within seconds to minutes of stress initiation [58,59], whereas it is possible that the subsequent results might be different in response to the age of PTMs or depending on treatment time. No genes were detected at 3 h, while the number of up-regulated genes induced by mechanical damage is slightly less than that induced by PTM infestation at 48 h and 96 h. This suggests that PTM feeding induced a faster and stronger signal than mechanical damage. Over-accumulation of ROS is toxic to plants, for which plants activate stringent ROS scavenging mechanisms, including antioxidant enzymes and non-enzymatic metabolites, to remove excess ROS and maintain normal levels of ROS [17,58,59,60,61,62]. Therefore, it is reasonable to see that there are more up-regulated genes related to ROS scavenging at 48 and 96 h after mechanical injury and PTM feeding, even though some were far more than down-regulated genes (Table 2). It has been observed that not only can ROS activate MAPK signaling, but also MAPK cascades can regulate ROS-related genes [17,60,61,62]. In our study, one and six MAPK-related up-regulated genes were observed at 3 h post PTM infestation and 96 h after mechanical damage, respectively, which may imply that MAPK cascades were activated at 3 h post PTM feeding, while MAPK signals were induced by mechanical damage after 48 h.

The activation of ROS signalings and MAPK cascades are usually accompanied by the rapid initiation of phytohormone networks and the complex interplay between ROS, MAPKs and hormones brings them to a level of mutual coordination [63,64,65]. In terms of JA biosynthesis, most genes were up-regulated under mechanical damage and PTM herbivory, which was largely consistent with the reported high accumulation of JA and JA–Ile under two treatments post one hour in Mao et al. study [20]. This further indicated that JA plays an essential role in mediating plant defenses against lepidopteran-chewing herbivores [66,67,68]. In our study, 13-LOX genes were more strongly induced than 9-LOX genes responsible for the production of 10-OPDA (Figure 5B). However, 9-lipoxygenase pathway is preferentially stimulated in cultured potato cells in response to treatment with P. infestans elicitor [69]. Feeding by S. frugiperda and beet armyworm Spodoptera exigua larvae on maize also induces the expression of 9-LOX genes to a greater extent than 13-LOX genes [70,71]. Species of herbivores/plants and different types of external stimuli could account for this disparity. Compared with MeJA, JA is inclined to be metabolized into JA–Ile as a major bioactive signal in the dynamic regulation of the JA signaling system [67,72], which is consistent with our results (Figure 5B). Moreover, we also identified several genes belonging to the GH3 family with the closest sequence homology to JAR1, but their role in JA metabolism is not known (Figure 5B). In Mao et al. study, accumulation of ABA was not induced significantly at 1 h by PTM actual herbivory and mechanical damage; even its induction was repressed by microbe from orally secreted bacteria (OSB) [20]. Most ABA biosynthesis genes were all down-regulated in CK-vs-M, CK-vs-P and M-vs-P comparisons in our study (Figure 7). The two research results emphasize the consistent performance of ABA-related genes in the potato’s defense against PTM feeding. Interestingly, unlike JA and ABA, the expression patterns of SA biosynthesis-related genes in CK96-vs-M96 and CK96-vs-P96 were different from that in M96-vs-P96 (Figure 6B). Accumulation of SA was also not induced significantly by all types of reorganized in a cumulative way potato tuber moth herbivory stimuli components in the Mao et al. study [20]. We previously mentioned that the M-vs-P groups possessed different proportions of genes from different potato tuber moth herbivorous stimuli (Figure 4B–D). Combining the above results, we speculated that mixed subsets of the genes from different potato tuber moth herbivory stimuli have different expression patterns in the synthesis of SA, which may result in the homeostasis of SA level. Ethylene biosynthesis-related genes were highly induced locally in M/CK, P/CK and P/M comparisons (Figure 8), which may imply ET pathway was involved in the defense against two treatments. In addition, we also detected the expression of all genes in the JA/SA/ABA/ETH signal transduction, which functions downstream from phytohormone production, and potato plants may have elevated constitutive expression of SA and ABA signaling cascade (Table S4).

A crosstalk between phytohormones depends on the plant–herbivore system has been studied. Many studies have reported that the combination of ET and JA can synergistically induce plant defense genes against biotic stress, including herbivores and pathogens [73,74,75]. In the direct defense of Nicotiana attenuata plants against chewing herbivores, jasmonic acid is a central mediator of defense gene expression, whose effect is modulated by ethylene [76]. Therefore, combining the research result of Mao et al. [20], we speculated that jasmonate and ethylene act in synergistic manners in the defense reaction of the potato to PTM feeding and mechanical damage, while salicylic acid and abscisic acid both play less significant roles in the defense response. ERF1 and EIN3 are downstream components of the ethylene signal transduction. It has been demonstrated that ERF1 is a key integrator of JA and ET signals, and its induction requires both signaling pathways simultaneously to be activated [77]. Zhu et al. speculated that EIN3/EIL1 is the direct molecular link for jasmonate-ethylene synergistic interactions based on the previously accumulated data [78]. In ET signal transduction, most genes, including the encoding ERF1 and EIN3/EIL were up-regulated; even the genes EIL3 (PGSC0003DMG400016747) and ERF1B (PGSC0003DMG400010285) had FC values exceeding 256 (Table S4). Moreover, ethylene plays an important accessory role in JA-mediated plant defenses against herbivores, and they can synergistically regulate the synthesis of downstream alkaloids etc., secondary metabolites [76,79], and the expression of protease inhibitors (PIs) genes [80]. From the number of up/down-regulated genes in the biosynthesis of various secondary metabolites and protease inhibitors, it can be concluded that the biosynthesis of terpenoids, alkaloids, steroids, and protease inhibitors in the potato is significantly induced by potato tuber moth feeding (Table 3 and Table 4). Flavonoids are derived from the phenylpropanoid pathway and belong to the phenolics. However, their biosynthesis was repressed by both treatments, which may result from the down-regulation of the genes encoding phenylalanine ammonia lyase (PAL), a key enzyme in this pathway (Figure 6). Serine protease inhibitors play a more prominent role in lepidopteran insects (Lepidoptera) [8], which is in line with our results (Table S11).

Transcription factor families have important regulatory roles in stress tolerance and mediating the expression of downstream defense genes in plants. Many excellent reviews and literature have reported that several transcription factor families, such as NAC, WRKY, AP2/ERF, bHLH, MYB, TCP, and bZIP are associated with biotic stress response in the potato [81]. The G2-like (GLKs) family plays an active role in resisting pathogen invasion, regulating leaf senescence and chloroplast development, and responding to abiotic stresses [82]. Only three up-regulated genes associated with the bZIP family appeared at 48 h after mechanical damage, whereas only five down-regulated genes were detected at 48 h after PTM infestation. Interestingly, the TCP family showed opposite gene expression patterns at 48 h after both treatments (Table S7), which indicated that the TFs families may fine-tune the response of plants to different external stimuli.

In solanaceae crops (e.g., potato and tobacco), the LecRK/LecRLK or NbLRK1 genes generate resistance by sensing elicitors produced by insects and pathogens [57,83]. The plant–pathogen interaction can affect the induction rapidity and effectiveness of chitinase [83]. In our study, the up-regulated genes related to plant–pathogen interaction, lectin receptor-like protein kinases (LecRLKs) and chitinases showed a consistent change in expression over time (Figure 10, Table 4). It has been shown that infestation by chewing Pieris rapae larvae induces resistance in Arabidopsis not only to *P. rapae* itself, but also to several microbial pathogens [84], indicating an overlap in the signaling network of plant defense against insects and pathogens, which is consistent with our results. This overlap suggests that the regulation of adaptive responses in plants is a delicate balance between protection against microbial and insect invaders [55]. De Vos et al. found that consistent changes in Arabidopsis transcriptional profiles caused by pathogens and insects with different attack modes not only showed considerable overlap but also varied in number with different combinations of attackers [54]. Additionally, Nilaparvata lugens or Chilo suppressalis infestation caused extensive up-regulation of genes related to phytopathogenic interactions in rice at 3 h and 6 h with a much higher proportion of up-regulated genes compared with our results [18]. Therefore, the magnitude of genetic overlap triggered by pathogens and insects reflects the intensity of the plant response, which is dependent on treatment time and plant-attacker combinations [54]. It would be interesting to further elucidate the role of uncharacterized genes such as protease inhibitors, chitinases, and lectins in plant–insect interactions in the future through gene overexpression, silencing, mutant analysis, etc.

## 4. Materials and Methods

### 4.1. Insect Colony

Potato tuber moth (*Phthorimaea operculella*, PTM) populations were collected from potato fields in Kunming, Yunnan Province, China and further raised for several generations on potato variety, Hezuo 88 (HZ 88). The obtained PTM eggs were placed in Petri dishes lined with moist filter paper and incubated in a growth chamber (temperature of 28 ± 2 °C, relative humidity 80%, photoperiod 12L:12D). Newly hatched PTM larvae were used for the experiments.

### 4.2. Plant Growth, Treatment and Sample Collection

Hezuo 88 healthy seed potatoes (only one bud eye per tuber was kept) were grown in plastic pots (diameter × height = 20 cm × 30 cm) and placed in a greenhouse (temperature 27 ± 2 °C, RH 75%, photoperiod 14L:10D) for cultivation. To avoid other insect attacks and interactions between plants, each plastic pot was placed 2 m apart and covered with 100-mesh nylon nets. The potato plants were regularly watered and fertilized according to the same criteria and were left to grow to five fully expanded leaves for the experiment. PTM larvae were starved for 6 h before being used to infest potatoes. One freshly hatched PTM larva at the age of 2 days was inoculated manually on five fully expanded leaves of potato, respectively, which was the PTM infestation treatment (P). The mechanical damage treatment (M) was achieved by randomly pricking the potato leaves obliquely at an angle of about 45 degrees with an alcohol-sterilized No. 3 insect needle. Potato plants without both treatments were used as a control (CK). At 3 h, 48 h, and 96 h post CK, M, and P treatments, potato leaves with uniform size and leaf age were harvested in aluminum foil and flash-frozen in liquid nitrogen for RNA extraction. For each treatment, three replicate samples at each time point were obtained from three uniformly growing and independent potato plants.

### 4.3. RNA Extraction, Illumina Library Construction and Sequencing

Total RNA was extracted from every sample using Trizol reagent kit (Invitrogen, Carlsbad, CA, USA) according to the manufacturer’s protocol and treated with RNase-free DNase I to remove genomic DNA contamination. RNA quality was assessed on an Agilent 2100 Bioanalyzer (Agilent Technologies, Palo Alto, CA, USA) and checked using RNase free agarose gel electrophoresis. After total RNA was extracted, eukaryotic mRNA was enriched by Oligo(dT) beads. Then, the enriched mRNA was fragmented into short fragments using a fragmentation buffer and reverse transcripted into cDNA with random primers. Second-strand cDNA was synthesized by DNA polymerase I, RNase H, dNTP and buffer. Then the cDNA fragments were purified with a QiaQuick PCR extraction kit (Qiagen, Venlo, The Netherlands), end-repaired, poly(A) added, and ligated to Illumina sequencing adapters. The cDNA library fragments were purified with the AMPure XP system to obtain cDNA fragments with a preferred around 200 bp, PCR amplified, and sequenced using Illumina HiSeq2500 by Gene Denovo Biotechnology Co. (Guangzhou, China).

### 4.4. Processing and Analysis of RNA-Seq Data

To obtain high-quality clean reads, raw reads were further filtered by fastp V0.18.0 [85] (https://github.com/OpenGene/fastp, accessed on 9 April 2023) to remove reads containing adapters, poly(N) and low-quality reads containing more than 50% of low-quality (Q-value ≤ 20) bases. An index of the potato reference genome was built, and paired-end clean reads were mapped to the reference genome SolTub_3.0 (https://www.ncbi.nlm.nih.gov/assembly/GCF_000226075.1, accessed on 10 April 2023) using HISAT2.2.4 [86] with “-rna-strandness RF” and other parameters set as a default. The mapped reads of each sample were assembled by using StringTie v1.3.1 [87,88] in a reference-based approach. For each transcription region, a FPKM (fragment per kilobase of transcript per million mapped reads) value was calculated to quantify its expression abundance and variations using StringTie software.

RNA differential expression analysis was performed by DESeq2 [89] software between two different groups (and by edgeR [90] between two samples). The Benjamini–Hochberg algorithm was used to adjust the *p*-value and hence control the false discovery rate (FDR). The genes with the parameter of false discovery rate (FDR) below 0.05 and absolute fold change ≥ 2 were considered differentially expressed genes (DEGs). Related genes between different treatment groups were identified based on gene annotation and differential expression analysis.

### 4.5. GO Functional and KEGG Pathway Enrichment Analysis

GO enrichment analysis provides all GO terms that are significantly enriched in DEGs compared to the genome background and filters the DEGs that correspond to biological functions. Firstly, all DEGs were mapped to GO terms in the Gene Ontology database (http://www.geneontology.org/, accessed on 20 April 2023), gene numbers were calculated for every term, and significantly enriched GO terms in DEGs compared to the genome background were defined by hypergeometric test. This analysis was able to recognize the main biological functions that DEGs exercise. Genes usually interact with each other to play roles in certain biological functions. Pathway-based analysis helps to further understand the genes’ biological functions. KEGG is the major public pathway-related database [90]. Pathway enrichment analysis identified significantly enriched metabolic pathways or signal transduction pathways in DEGs compared with the whole genome background.

The calculated *p*-values were processed through FDR Correction, taking FDR ≤ 0.05 as a threshold. GO terms and pathways meeting this condition were defined as significantly enriched GO terms and pathways in DEGs, respectively.

## 5. Conclusions

In conclusion, the defense mechanism of the potato is activated after the initiation of mechanical damage and PTM infestation, and response to two treatments built up over time.

GO functional enrichment showed PTM feeding and mechanical damage activated primary and secondary metabolism over time and suppressed the growth and development of the potato. Phytohormone analysis showed that genes involved in JA and ET signaling pathways were strongly induced, and both may synergistically induce defense responses against herbivores, with JA having a more dominant role than ET. At 48 h and 96 h, more up-regulated genes linked to ROS scavenging were observed, as were the genes encoding secondary metabolites. This suggests that the antioxidant system plays an indispensable role in plant adaptation to both stimuli, while the production of secondary metabolites such as terpenoids, alkaloids, and steroids would presumably benefit only PTM-challenged plants from the proportion of up- and down-regulated genes. Several TF families, such as bHLH, AP2/EREBP, WRKY and MYB, were differentially regulated, while the Trihelix family and the G2-like family appeared to be particularly active in mediating the response to mechanical damage and PTM infestation, respectively. Both treatments also induced gene expression of many pathogenesis-related (PR) proteins (e.g., protease inhibitors and chitinases) and lectins.

Our exploration of mechanical damage and actual PTM infestation at multiple time points makes a strong addition to the gene expression patterns of potato–PTM interactions over the time span.

## Figures and Tables

**Table 1:** DEGs associated with reactive oxygen species (ROS) generating.

Category		AO	GOX	HPPD	GAOX	ND	COX	RBOH	SOD	FIN4	QSOX	ACX	AAO	ERO	FMO	Total
CK3-vs-M3	up	0	0	0	0	0	0	0	0	0	0	0	0	0	0	0
	dw	0	0	0	0	0	0	0	0	0	0	0	0	0	0	0
CK48-vs-M48	up	1	0	0	2	1	1	0	1	1	0	0	0	2	0	9
	dw	0	1	0	1	0	0	1	0	0	0	1	0	0	0	4
CK96-vs-M96	up	0	0	0	1	0	0	2	0	0	0	4	1	0	0	8
	dw	4	0	1	3	0	2	1	0	1	0	0	0	0	0	12
M/CK Total	up	1	0	0	3	1	1	2	1	1	0	4	1	2	0	17
	dw	4	1	1	4	0	2	2	0	1	0	1	0	0	0	16
CK3-vs-P3	up	0	0	0	2	4	0	0	1	0	2	0	1	0	1	11
	dw	4	1	1	1	0	0	2	0	0	0	0	0	0	1	10
CK48-vs-P48	up	3	0	0	1	0	0	3	1	0	0	2	1	0	1	12
	dw	2	0	1	4	1	2	2	0	0	0	0	1	0	0	13
CK96-vs-P96	up	1	0	1	3	0	0	1	0	0	0	5	2	0	0	13
	dw	3	0	1	3	1	2	2	1	1	0	0	0	0	0	14
P/CK Total	up	4	0	1	6	4	0	4	2	0	2	7	4	0	2	36
	dw	9	1	3	8	2	4	6	1	1	0	0	1	0	1	37
M3-vs-P3	up	1	1	0	4	3	0	0	1	1	1	1	1	0	2	16
	dw	7	1	1	0	1	0	2	0	0	0	0	0	0	2	14
M48-vs-P48	up	5	1	0	1	2	0	4	3	0	0	0	1	0	1	18
	dw	5	0	1	3	1	5	1	1	1	0	0	1	1	0	20
M96-vs-P96	up	4	0	0	2	0	0	0	0	0	0	1	1	0	0	8
	dw	1	0	0	0	0	0	0	1	0	0	0	0	0	0	2
P/M Total	up	10	2	0	7	5	0	4	4	1	1	2	3	0	3	42
	dw	13	1	2	3	2	5	3	2	1	0	0	1	1	2	36

Up—up-regulated genes; dw—down-regulated genes; AO—amine oxidase; GOX—glycolate oxidase; HPPD—4-hydroxypheny lpyruvate dioxygenase; GAOX—gibberellin dioxygenase; ND—NADH dehydrogenase; COX—cytochrome c oxidase subunit; RBOH—respiratory burst oxidase homolog; SOD—superoxide dismutase; FIN4—L-aspartate oxidase; QSOX—sulfhydryl oxidase; ACX—acyl-CoA oxidase; AAO—aldehyde oxidase; ERO—ER oxidoreductin; FMO—flavin-containing monooxygenase.

**Table 2:** DEGs associated with reactive oxygen species (ROS) scavenging.

Category		CAT	APX	POD	Fd	TRX	GRX	GST	MDHAR	DHAR	GPX	PrxR	NRX	Total
CK3-vs-M3	up	0	0	0	0	0	0	0	0	0	0	0	0	0
	dw	0	0	0	0	0	0	0	0	0	0	0	0	0
CK48-vs-M48	up	0	1	3	3	1	0	5	0	1	0	0	0	14
	dw	0	1	2	0	2	3	2	0	0	0	0	0	10
CK96-vs-M96	up	1	0	6	1	5	6	0	0	0	1	0	0	20
	dw	1	3	5	3	10	4	10	0	0	1	1	1	39
M/CK Total	up	1	1	9	4	6	6	5	0	1	1	0	0	34
	dw	1	4	7	3	12	7	12	0	0	1	1	1	49
CK3-vs-P3	up	0	0	3	1	4	0	2	0	2	0	1	0	13
	dw	0	3	1	2	2	8	6	0	0	0	0	0	22
CK48-vs-P48	up	0	1	22	4	2	15	0	1	1	1	4	0	51
	dw	1	2	1	3	4	5	10	0	0	0	0	1	27
CK96-vs-P96	up	1	0	22	1	5	14	1	0	0	1	1	0	46
	dw	1	1	5	2	8	2	7	0	0	0	1	0	27
P/CK Total	up	1	1	47	6	11	29	3	1	3	2	6	0	110
	dw	2	6	7	7	14	15	23	0	0	0	1	1	76
M3-vs-P3	up	0	0	3	3	5	0	2	0	1	0	1	0	15
	dw	1	2	9	3	10	14	24	0	0	0	0	1	64
M48-vs-P48	up	1	3	24	3	3	11	2	1	1	0	5	0	54
	dw	1	2	2	3	8	4	17	0	0	0	0	1	38
M96-vs-P96	up	0	0	15	3	2	9	2	0	1	0	0	0	32
	dw	0	0	0	0	0	3	0	0	0	0	0	0	3
P/M Total	up	1	3	42	9	10	20	6	1	3	0	6	0	101
	dw	2	4	11	6	18	21	41	0	0	0	0	2	105

Up—up-regulated genes; dw—down-regulated genes; CAT—catalase isozyme; APX—ascorbate peroxidase; POD—peroxidase; Fd—ferredoxin; TRX—thioredoxin; GRX—glutaredoxin; GST—glutathione s-transferase; MDHAR—monodehydroascorbate reductase; DHAR—dehydroascorbate reductase; GPX—glutathione peroxidase; PrxR—peroxiredoxin; NRX—nucleoredoxin.

**Table 3:** DEGs associated with plant secondary metabolites biosynthesis.

Category		Terpenoids	Flavonoids	Alkaloids	Steroids	Quinone	Total
CK3-vs-M3	up	2	0	0	0	0	2
	dw	0	0	0	0	0	0
CK48-vs-M48	up	5	5	0	0	1	11
	dw	10	8	2	0	2	22
CK96-vs-M96	up	12	2	7	7	2	30
	dw	28	23	16	8	10	85
CK3-vs-P3	up	9	2	6	5	2	24
	dw	18	13	11	3	5	50
CK48-vs-P48	up	12	6	15	4	3	40
	dw	24	16	9	4	6	59
CK96-vs-P96	up	24	6	12	9	4	55
	dw	23	21	13	5	10	72
M3-vs-P3	up	15	7	6	9	2	39
	dw	27	24	18	4	6	79
M48-vs-P48	up	16	8	20	7	5	56
	dw	24	11	8	3	7	53
M96-vs-P96	up	12	12	8	6	4	42
	dw	0	0	0	0	0	0

Up—up-regulated genes; dw—down-regulated genes.

**Table 4:** DEGs involved in proteinase inhibitors (PIs), chitinases and MAPK cascades.

Category		M3/CK3	M48/CK48	M96/CK96	P3/CK3	P48/CK48	P96/CK96	P3/M3	P48/M48	P96/M96	Total
PIs	up	0	9	1	5	8	7	10	4	6	50
	dw	0	1	0	1	0	0	1	3	0	6
Chitinases	up	0	1	3	1	2	6	2	7	4	26
	dw	0	2	5	7	3	1	11	4	0	33
MAPK	up	0	0	6	1	1	6	2	2	1	19
	dw	0	0	6	0	4	6	2	4	1	23

M3/CK3—abbreviation for CK3-vs-M3. The abbreviated forms of other comparisons as described for the first comparison.

## Data Availability

The transcriptomics dataset is available under Bioproject: PRJNA978946.

## Supplementary Materials

The following supporting information can be downloaded at: https://www.mdpi.com/article/10.3390/plants12173092/s1, Table S1: Sunmary of RNA-Seq data; Table S2: The top 20 GO enrichment terms in each comparison group; Table S3: The top 20 KEGG enrichment pathways in each comparison group; Table S4: The KEGG enrichment pathways of up- and down-regulated genes in each comparison group; Table S5: DEGs involved in the phytohormones pathways; Table S6: DEGs involved in the reactive oxygen species; Table S7: Number of DEGs involved in transcription factors; Table S8: The KEGG enrichment pathways of up- and down-regulated TFs genes in each comparison group; Table S9: DEGs involved in secondary metabolites; Table S10: DEGs involved in plant–pathogen interaction; Table S11: DEGs involved in PI, chitinase, lectin and MAPK; Table S12: The GO enrichment terms of up- and down-regulated genes associated with plant-pathogen interactions in each comparison group; Table S13: The GO enrichment terms of up- and down-regulated genes associated with LecRLKs in each comparison group; Figure S1: Differentially expressed genes (Padj < 0.05 and an absolute value of Log_2_FoldChange (relative to the control) ≥2 was used as the standard for screening DEGs).
