# Supplementary material for: Comparative Transcriptome Analysis of Defense Response of Potato to Phthorimaea operculella Infestation
Source: Plants (Basel). 2023 Aug 29;12(17):3092. doi: 10.3390/plants12173092 (PMC10490199; doi:10.3390/plants12173092)
Supplement: Supplementary file 1 [file plants-12-03092-s001.zip › enrichment analyses/Figure S1 Differentially expressed genes.pdf]

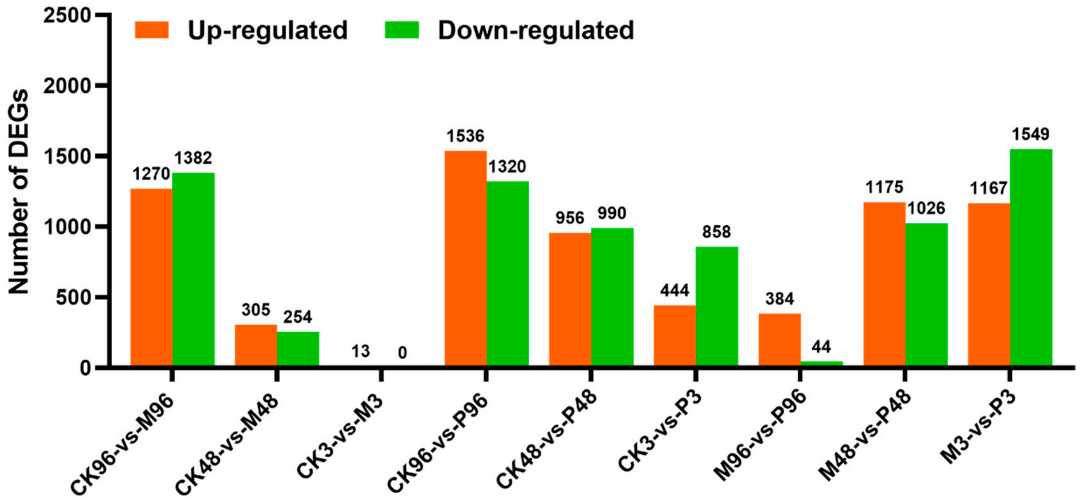

Figure S1. Number of up- and down-regulated differentially expressed genes (DEGs) among different comparisons at each time point. Note: Padj < 0.05 and an absolute value of Log2FoldChange (relatived to the control) ≥2 was used as the standard for screening DEGs.

To understand the transcriptional changes in potato leaves after mechanical damage, transcription profiles of the mechanical wounding and control plants for 3h, 48h and 96h were compared. Padj < 0.05 and an absolute value of Log_2FoldChange (relatived to the control) ⩾2 was used as the standard for screening DEGs. A total of 559 (305 up and 254 down) and 2652 (1270 up and 1382 down) DEGs were found for the comparisons: CK48-vs-M48 and CK96-vs-M96, respectively, whereas 13 up DEGs induced only within CK3-vs-M3 comparison (Figure S1).

The transcriptomes of PTM infestation and undamaged control were compared to study the transcriptional changes iuduced by potato tuber moth feeding. The data exhibited 1302 (444 up and 858 down), 1946 (956 up and 990 down) and 2856 (1536 up and 1320 down) DEGs after 3h, 48h and 96h, respectively (Figure S1).

A total number of 12206 DEGs were altered, of which 2716 (1167 up and 1549 down), 2201 (1175 up and 1026 down) and 428 (384 up and 44 down) DEGs were detected respectively for 3h, 48h and 96h by comparing the transcription profiles of PTM-infested and mechanically damaged plants (Figure S1).
