# Supplementary material for: Comparative Transcriptome Analysis of Defense Response of Potato to Phthorimaea operculella Infestation
Source: Plants (Basel). 2023 Aug 29;12(17):3092. doi: 10.3390/plants12173092 (PMC10490199; doi:10.3390/plants12173092)
Supplement: Supplementary file 1 [file plants-12-03092-s001.zip › Table S1 Sunmmary of RNA-Seq data.pdf]

Table 1 Summary table of RNA-Seq data in potato leaves

Samples	Condition	RawData	CleanData(%)	Total_mapped(%)	Unique_mapped(%)	GC(%)	Q30(%)
M96-1	Mechanically wounded 96h	37707648	37676036 (99.92%)	16500227 (85.40%)	15778037 (81.66%)	48.15	94.09
M96-2	Mechanically wounded 96h	45922498	45869590 (99.88%)	21053536 (86.01%)	20150808 (82.33%)	47.08	92.86
M96-3	Mechanically wounded 96h	46768008	46714340 (99.89%)	14524511 (81.89%)	14048153 (79.21%)	47.89	93.05
M48-1	Mechanically wounded 48h	44280402	44222018 (99.87%)	36912137 (89.43%)	35348404 (85.64%)	45.26	92.99
M48-2	Mechanically wounded 48h	37271202	37224532 (99.87%)	30191224 (88.64%)	29003286 (85.16%)	45.17	93.00
M48-3	Mechanically wounded 48h	45901504	45844272 (99.88%)	38897352 (88.05%)	37499435 (84.89%)	44.37	92.89
M3-1	Mechanically wounded 3h	41353642	41298248 (99.87%)	36457874 (91.10%)	34627575 (86.53%)	45.19	93.55
M3-2	Mechanically wounded 3h	38712098	38656650 (99.86%)	33930587 (90.71%)	32142822 (85.93%)	44.97	92.86
M3-3	Mechanically wounded 3h	43119864	43059952 (99.86%)	37577840 (91.12%)	35460267 (85.98%)	45.29	93.24
P48-1	PTM infested 48h	42299580	42234264 (99.85%)	36464478 (87.84%)	34703464 (83.60%)	44.62	92.41
P48-2	PTM infested 48h	51584672	51477964 (99.79%)	44293524 (89.08%)	42138355 (84.75%)	44.84	91.08
P48-3	PTM infested 48h	49901750	49842562 (99.88%)	26797724 (89.62%)	25384839 (84.89%)	46.59	93.47
P96-1	PTM infested 96h	43643080	43594140 (99.89%)	36330322 (86.65%)	34681035 (82.72%)	44.92	93.36

P96-2	PTM infested 96h	41652304	41607824 (99.89%)	17893637 (86.59%)	17113035 (82.81%)	47.31	93.22
P96-3	PTM infested 96h	39238544	39179152 (99.85%)	33806616 (89.26%)	32404891 (85.56%)	44.52	92.87
P3-1	PTM infested 3h	45266476	45202030 (99.86%)	38317436 (89.41%)	36684882 (85.60%)	44.74	92.99
P3-2	PTM infested 3h	44177308	44111112 (99.85%)	37953309 (90.05%)	36329083 (86.19%)	45.00	93.42
P3-3	PTM infested 3h	44838608	44777686 (99.86%)	39005195 (89.86%)	37372634 (86.10%)	44.68	92.48
CK3-1	Non-infested Control 3h	39460346	39406094 (99.86%)	34583804 (90.46%)	32799283 (85.79%)	44.98	92.57
CK3-2	Non-infested Control 3h	51880920	51836050 (99.91%)	45869528 (91.75%)	42974467 (85.96%)	46.07	93.61
CK3-3	Non-infested Control 3h	41018148	40950074 (99.83%)	33625251 (84.44%)	32027461 (80.43%)	45.05	92.23
CK48-1	Non-infested Control 48h	39152258	39104126 (99.88%)	34047057 (89.70%)	32780601 (86.36%)	44.65	92.64
CK48-2	Non-infested Control 48h	44322740	44274964 (99.89%)	38202593 (89.53%)	36643663 (85.87%)	44.93	92.81
CK48-3	Non-infested Control 48h	45283274	45234938 (99.89%)	39183555 (89.73%)	37683121 (86.29%)	44.70	92.80
CK96-1	Non-infested Control 96h	37576124	37532408 (99.88%)	32603197 (90.38%)	31338908 (86.87%)	45.03	92.92
CK96-2	Non-infested Control 96h	48675922	48630226 (99.91%)	42739893 (90.43%)	40700631 (86.11%)	45.05	93.44
CK96-3	Non-infested Control 96h	43034396	42983732 (99.88%)	37378883 (89.91%)	35759623 (86.01%)	45.23	92.64

Note: RawData, the number of reads in the original data; CleanData (%), the number and proportion

of reads after raw data filtering in each sample; Total_mapped (%), the number and proportion of reads matched to potato genome.
